# Inflammatory Reaction in Neurological Diseases

**DOI:** 10.1155/2014/408514

**Published:** 2014-12-31

**Authors:** Hung-Chen Wang, Cheng-Hsien Lu, Kuang-I Cheng, Jason Cheng-Hsuan Chiang

**Affiliations:** ^1^Department of Neurosurgery, Kaohsiung Chang Gung Memorial Hospital, Chang Gung University College of Medicine, Kaohsiung 83301, Taiwan; ^2^Department of Neurology, Kaohsiung Chang Gung Memorial Hospital, Kaohsiung 83301, Taiwan; ^3^Department of Biological Science, National Sun Yat-Sen University, Kaohsiung 80424, Taiwan; ^4^Department of Anesthesiology, Kaohsiung Medical University Hospital, Kaohsiung 807, Taiwan; ^5^Department of Neuropathology, University of Pittsburgh Medical Center, Pittsburgh, PA 15213, USA

Inflammatory reaction in the central nervous system (CNS) is now recognized to be a feature of all neurological disorders. In neurological degenerative diseases, such as Parkinson's disease (PD) and Alzheimer's disease (AD), there is prominent infiltration of various leukocyte subsets into the CNS or there is intense activation of microglia with resultant elevation of many inflammatory mediators within the CNS. In acute critical CNS diseases, such as delayed deterioration associated with vasospasm after subarachnoid hemorrhage (SAH), ischemic stroke, spontaneous intracerebral hemorrhage (ICH), and traumatic brain injury (TBI), recent evidences show that inflammation may be a potential target for therapy. Inflammation has become a promising area of research for new treatments. To accelerate the process of translating this information to clinical applications, a number of important issues must be addressed such as their ability to consistently detect characteristic cerebral deficits in individuals with neurological degenerative diseases, the relationships of cerebral injuries to clinical symptoms and genetic characteristics, and the degree to which these injuries respond to different therapies. In this special issue, several research groups report findings that address some of these issues.

In neurological degenerative diseases, the paper by C. Millington et al. reviewed the role of chronic neuroinflammation in the pathogenesis of* Alzheimer's disease* (AD). With the glial fibrillary acidic protein-interleukin 6 (GFAP-IL6) transgenic mice model, the authors found that this animal model, in which chronic neuroinflammation triggered constitutive expression of the cytokine interleukin-6 (IL-6) in astrocytes, could serve as an excellent tool for drug discovery and validation in vivo. The paper by X. Su and H. J. Federoff reviewed the role of inflammation in* Parkinson's disease* (PD) neuropathology. They provide an overview of current knowledge on the temporal profile of central and peripheral immune responses in PD and discuss the potential synergistic effects of the central and peripheral inflammation in disease development. The study by W.-C. Lin et al. used TRODAT-1 SPECT to evaluate leukocyte apoptosis in PD patients and its association with central dopamine neuron loss. The leukocyte apoptosis and striatal dopamine transporter uptake ratios were further associated with increased severity and longer duration of disease. The interaction between brain and systemic inflammation may be responsible for the neurodegenerative disease progression. The paper by K. Lu et al. used the Longitudinal Health Insurance Database 2000 (LHID2000) to investigate and compare the risk of* dementia* between patients clinically diagnosed with* autoimmune rheumatic diseases* (ARD) and non-ARD patients during a 5-year follow-up period. Their findings suggest that patients with and without ARD were found to have similar risks of developing dementia.

In acute critical CNS diseases, the study by K.-W. Wang et al. used* traumatic brain injury* (TBI) model to determine whether simvastatin combined with an antioxidant could attenuate cerebral vascular endothelial inflammatory response after traumatic brain injury in rat. Their findings support that simvastatin combined with an antioxidant could provide neuroprotection and it may be attributed to a dampening of cerebral vascular endothelial inflammatory response. The study by W. Winardi et al. used a structural equation modeling to evaluate the predictive value of admission Glasgow Coma Scale (GCS) scores, duration of unconsciousness, neurosurgical intervention, and countercoup lesion on the impairment of memory and processing speed functions six months after a TBI. The study demonstrated that admission GCS score is a robust predictor of memory/processing speed dysfunctions after TBI. The study by N.-W. Tsai et al. investigated serum thiobarbituric acid-reactive substances (TBARS) and free thiol levels in different subtypes of* acute ischemic stroke* (AIS) and evaluated their association with clinical outcomes. They found that patients with large-vessel disease have higher oxidative stress but lower antioxidant defense compared to those with small-vessel disease after AIS. Serum TBARS level at the acute phase of stroke is a potential predictor for three-month outcome. And the paper by C.-M. Su et al. aimed to determine whether serum adhesion molecules are associated with* septic encephalopathy* (SE). Their findings demonstrate that SE implies higher mortality in nontraumatic, nonsurgical patients with severe sepsis. Serum vascular cell adhesion molecule-1 (VCAM-1) level on presentation is a more powerful predictor of SE in these patients than lactate concentration and other adhesion molecules on admission.

In the CNS malignancy, the study by D. Winardi et al. investigated the relationship between protein expressions of two autophagy markers, LC3B and Beclin-1, with clinical parameters in* astrocytoma patients*. Their results suggest that astrocytoma cancer stem-like cells together with enhanced autophagy may cause resistance to radiation therapy/chemotherapy and that targeting the cancer stem-like cell in astrocytoma may offer a viable therapeutic approach. And the study by C.-L. Chung et al. investigated DAPK protein expression and promoter hypermethylation in* central neurocytoma and oligodendroglioma*. Their results show that DAPK promoter hypermethylation and repressed expression of DAPK protein were more common in central neurocytoma than in oligodendroglioma. Thus, DAPK promoter hypermethylation could be useful for differential diagnosis between these two types of tumors.

In summary, the papers in this series highlight several important research strategies that are making it increasingly evident that the neuroinflammation is of translational value for different types of neurological diseases. The results from these studies not only help us to understand the pathogenesis of these disorders but also show great potential to provide urgently needed objective biomarkers for clinical diagnosis and evaluation. Knowledge and understanding of these conditions have led to the development of animal models, successful therapies, and novel tools to characterize these clinical conditions and provide better care to patients.

*Hung-Chen Wang* *Cheng-Hsien Lu* *Kuang-I Cheng* *Jason Cheng-Hsuan Chiang*

